# Computed tomography versus lower endoscopy as initial diagnostic method for evaluating patients with hematochezia at emergency room

**DOI:** 10.1097/MD.0000000000020311

**Published:** 2020-05-29

**Authors:** Hee Sung Lee, Sun Hyung Kang, Woo Sun Rou, Hyuk Soo Eun, Jong Seok Joo, Ju Seok Kim, Eaum Seok Lee, Hee Seok Moon, Seok Hyun Kim, Jae Kyu Sung, Byung Seok Lee, Hyun Yong Jeong

**Affiliations:** Division of Gastroenterology, Department of Internal medicine, Chungnam National University School of Medicine, Daejeon, Korea.

**Keywords:** colonoscopy, diagnostic performance, lower gastrointestinal bleeding, sigmoidoscopy

## Abstract

For acute lower gastrointestinal bleeding (LGIB), lower gastrointestinal endoscopy is the preferred initial diagnostic test. However, it is difficult to perform urgently. Computed tomography (CT) is a convenient alternative.

This study aimed to determine the diagnostic performance of CT compared to lower endoscopy as an initial test for evaluating acute LGIB.

The medical records of 382 patients who visited our emergency department with hematochezia between January 2012 and January 2017 were retrospectively analyzed. Of them, 112 underwent CT, 65 underwent colonoscopy, and 205 underwent sigmoidoscopy as an initial test. For each method, sensitivity, specificity, positive predictive value, and negative predictive value were calculated upon active bleeding site detection and LGIB etiology diagnosis.

The sensitivity, specificity, positive predictive value, and negative predictive value of CT for active bleeding site detection were 85.7%, 100%, 100%, and 96.9%, respectively, while those for identifying the etiology of LGIB were 87.4%, 40.0%, 83.5, and 47.6%, respectively.

CT was not inferior to lower endoscopy for active bleeding site detection. Early localization and the exclusion of active bleeding were possible with CT. Etiology was diagnosed with high sensitivity and PPV by CT. Thus, CT can be an alternative initial diagnostic tool for evaluating acute LGIB.

## 1 Introduction

Lower gastrointestinal bleeding (LGIB) is responsible for 20% to 40% of all gastrointestinal (GI) bleeding cases.^[1–3]^ Hematochezia is a common presentation of LGIB.^[2]^ Unlike upper GI bleeding (UGIB), most LGIB stop spontaneously with only supportive care.^[1–4]^ The mortality rate of LGIB has decreased over the past few decades and is reportedly 2% to 4%.^[3,4]^ Death is usually a consequence of underlying diseases rather than bleeding.^[3]^

Although LGIB tends to show a favorable clinical course, it is still associated with considerable morbidity and mortality in old populations, especially when accompanied by significant comorbities.^[1,2]^ Rebleeding may occur in 25% 46% of LGIB cases.^[5,6]^ Uncontrolled ongoing bleeding carries the risk of hemorrhagic shock, in which the mortality rate may increase to as high as 23%.^[7–9]^ Therefore, proper management is essential, especially in high-risk populations.

Recommendations have suggested that the standard initial diagnostic procedure for acute LGIB is colonoscopy or sigmoidoscopy for hemodynamically stable patients and upper endoscopy for initially unstable patients or when UGIB is suspected.^[1–3]^ Colonoscopy has been considered the first-line diagnostic tool due to its high diagnostic yield and ability to simultaneously provide therapeutic procedures.^[1–3,10,11]^

However, lower endoscopy is not easy for both patients and physicians, especially in emergency settings. Bowel preparation is a distressing experience for patient and may delay the diagnosis by up to several hours.^[12,13]^ If not properly done, entry to the upper level would be difficult with an increased risk of colorectal perforation.^[14,15]^ Even after the preparation, excessive blood clots and remaining fecal materials can prevent identification of the bleeding site and bleeding control. Moreover, endoscopy can only be performed when the patient is hemodynamically stable and cooperative, and on-duty skilled endoscopists must be available. In addition, the small-bowel bleeding site cannot be diagnosed by lower endoscopy, which is responsible for about 5% of all sources of GI bleeding.^[1–3,16,17]^

Computed tomography (CT) has gained attention as an accurate and convenient alternative for initial diagnosis of acute LGIB. With the development of multi-detector row CT (MDCT), this fast, safe, and non-invasive modality has shown promising results for the evaluation of LGIB.^[18–28]^ In a prospective study comparing MDCT and endoscopy for diagnosing the cause of acute LGIB, the sensitivity of MDCT for bleeding site detection and etiology identification were reportedly 100% and 88.2%, exceeding the diagnostic performance of endoscopy.^[18]^

Considering the self-limiting nature and favorable prognosis of most cases of LGIB, as long as active bleeding is initially ruled out by CT, we may safely plan for elective colonoscopy. In cases of active bleeding, early localization of bleeding site by initial CT would allow targeted therapy.^[29]^ Guidelines have recently started to include the CT angiography as a front-line diagnostic tool for evaluating acute LGIB^[29,30]^

However, evidence is currently limited regarding the routine use of CT instead of lower endoscopy in the initial management of LGIB. This study aimed to determine the whether CT can be used as an alternative initial diagnostic method instead of lower endoscopy for evaluating acute LGIB patients visiting the emergency room with the chief complaint of hematochezia.

## 2 Materials and methods

### 2.1 Study design

This retrospective study was conducted at a single tertiary care academic center and aimed to determine the diagnostic performance of triple-phase dynamic contrast-enhanced abdominopelvic MDCT compared to colonoscopy and sigmoidoscopy as an initial diagnostic tool for evaluating acute LGIB. The primary goal was to verify if initially performed CT can detect the active bleeding site as accurately as lower endoscopy. The secondary goal was to verify if initially performed CT can diagnose the etiology of LGIB as accurately as lower endoscopy.

Acute LGIB was defined in our study as GI bleeding presenting as hematochezia and originating from a lesion below the ligament of Treitz. Although recent studies have reclassified small bleeding as middle GI bleeding separately and defined LGIB as bleeding originating from either colon or rectum, for the purposes of this study, traditional definition of LGIB was used.

### 2.2 Patient selection

The medical records of patients who visited the emergency department of Chungnam National University Hospital between January 2012 and January 2017 with hematochezia as a chief complaint were retrospectively searched. Patients older than 18 years of age were eligible for inclusion. The following patients were excluded:

(1) those who did not undergo any diagnostic studies or refused to undergo further evaluations;
(2) those in whom UGIB was confirmed by esophagogastroduodenoscopy or CT;
(3) those with post-interventional bleeding;
(4) those with hemorrhoid bleeding; and
(5) those who underwent CT not meeting the criteria of triple-phase dynamic abdominopelvic MDCT

### 2.3 Data collection and evaluation

Enrolled acute LGIB patients were divided into 3 groups:

(1) sigmoidoscopy;
(2) colonoscopy; or
(3) triple-phase dynamic abdominopelvic MDCT as an initial diagnostic examination.

The initial method was selected by the gastroenterologist or emergency medicine physician on duty and the decision was made based on the patient's clinical presentation and availability of modalities. The endoscopist and radiologist on duty were available 24 hours a day. The on-duty radiologist reviewed the CT results upon request.

**Figure 1:**
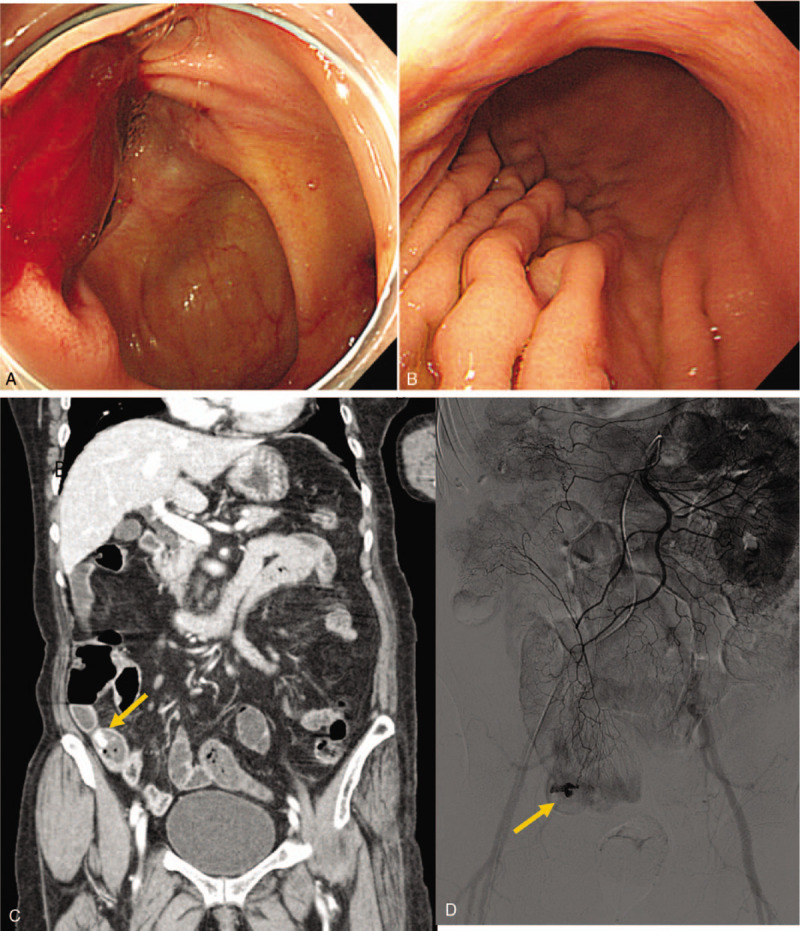
79-year-old female with hematochezia. A. initially performed colonoscopy revealing bleeding from the ileum, B. esophagogastroduodenoscopy showing non-specific finding, C. portal phase computed tomography showing active bleeding at the distal ileum with uncertain primary cause. D. angiography confirming the active bleeding. E. selective glue embolization was subsequently performed.

When the initial diagnosis was made but not definite, the result was verified by another diagnostic modality or gold standard test as a confirmatory test to evaluate its accuracy. Confirmatory tests included sigmoidoscopy, colonoscopy, CT, mesenteric angiography, capsule endoscopy, surgery, and repeat of the initial diagnostic test (Fig. 1).

If active bleeding was found by the initial test and confirmed by the confirmatory test or if certain etiology was initially diagnosed and the confirmatory test revealed the same diagnosis, the initial diagnosis was considered “true positive (TP).” If active bleeding was not found by the initial test and verified to be true by the confirmatory test or if the etiology of LGIB was not diagnosed by the initial and confirmatory tests, the initial diagnosis was considered “true negative (TN).” If the active bleeding site was found on the initial test but not on the confirmatory test or if the initial test revealed the certain etiology but the confirmatory test revealed no etiology or a different etiology, then the initial diagnosis was considered “false positive (FP).” If the initial test findings were negative but a further confirmatory test revealed the active bleeding site or if the etiology was not diagnosed by the initial test but was diagnosed on the confirmatory test, then the initial diagnosis was considered “false negative (FN).” If the diagnosis made by the initial test was absolutely definite and the bleeding completely stopped after treatment of the initially diagnosed bleeding source, a confirmatory test was not performed, and the initial diagnosis was regarded as TP.

On endoscopy, active bleeding was defined as spurting or oozing bleeding. An active bleeding site on CT was defined as the presence of extravasation of the contrast medium into the GI lumen on any of the enhanced phase images.

Using the TP, TN, FP, and FN values obtained by the above process, each initial diagnostic test's sensitivity, specificity, positive predictive value (PPV), and negative predictive value (NPV) when detecting the active bleeding site and identifying the etiology of LGIB were calculated to assess the diagnostic performance of each test. In addition, mean hospital stay and mean period time to the final diagnosis were measured for each test.

### 2.4 CT protocol

A 64-channel multidetector CT scanner (SOMATOM Sensation 64; Siemens Healthcare GmbH, Erlangen, Germany) was used to collect the abdominopelvic CT images during the entire study period. Our study only included the triple-phase dynamic contrast-enhanced abdominopelvic MDCT as an initial CT protocol which consisted of non-enhanced, arterial, and portal phases. The spiral scans were obtained with 64 × 0.6-mm collimation, 3-mm slice width, 0.5-second rotation time, 1.05 scan pitch, and 120 kV.

After non-enhanced pre-phase images were acquired, radiocontrast agent (iopamidol, dose 2.0 cc/kg, concentration 300 mg/mL) was injected through an intravenous catheter placed at the antecubital fossa or forearm. The infusion rate was 2.5% to 3.5 cc/s. Oral contrast media was not used. Unenhanced images were acquired first; after the contrast media injection, arterial and portal phase images were acquired 10 and 60 seconds after the abdominal aorta enhancement reaching 100 Housefield units. Axial plane images were constructed first and reconstructed into coronal and sagittal plane images on multiplanar reconstruction. The scan covered the area from diaphragm to the pubic symphysis.

Our institution operates abdominopelvic CTA protocol separately, which requires 3.5 to 4.0 cc/s of contrast media injection rate and includes 3-dimentional reconstruction of the vessels with the volume rendering technique. CTA was rarely used for the evaluation of the GI bleeding, but was usually performed for vascular injuries and traumas. CTA was not included in this study.

### 2.5 Statistical analysis

Continuous variables are reported as mean and standard deviation and compared using analysis of variance. Categorical variables are reported as number of patients and percentage and compared using the Chi-squared test. The diagnosis made by the initial diagnostic methods was compared to the final definite diagnosis and classified as TP, TN, FP, or FN. Contingency tables (2 × 2) were constructed for each of 3 initial tests, and sensitivity, specificity, PPV, and NPV were calculated from TP, TN, FP, and FN to evaluate the diagnostic accuracy. Confidence intervals (95%) were calculated using the Wilson score method with continuity correction.^[31]^ The McNemar test was used to check for significant differences between the initial diagnosis and final definite diagnosis. *P* values < .05 indicated statistically significant differences. All statistical analyses were performed using SPSS Statistics version 25 (International Business Machines Corp., Armonk, NY).

### 2.6 Ethics statement

The Institutional Review Board (IRB) of Chungnam National University Hospital approved this study (IRB number: 2018–05–045) and waived the need for written participant consent due to the study's retrospective design.

## 3 Results

**Figure 2:**
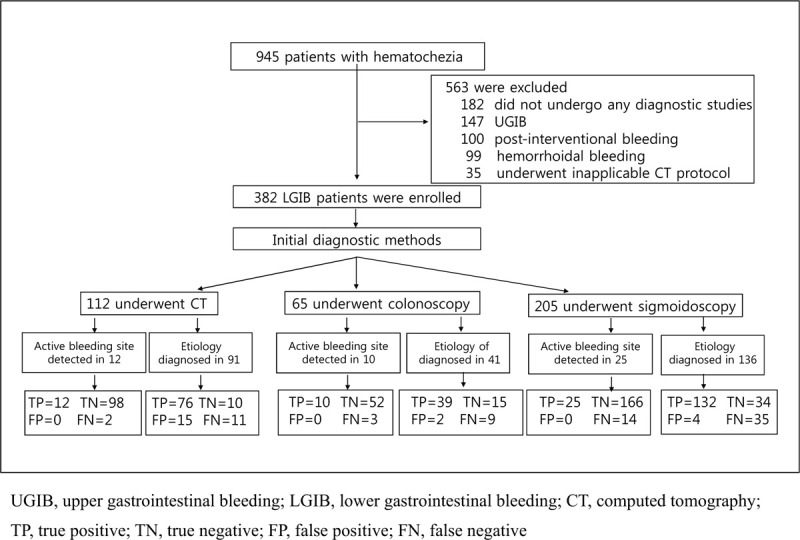
Flow diagram.CT = computed tomography, FN = false negative, FP = false positive, LGIB = lower gastrointestinal bleeding, TN = true negative, TP = true positive, UGIB = upper gastrointestinal bleeding.

From January 2012 to January 2017, a total of 945 patients who visited our emergency department with the chief complaint of hematochezia were assessed for eligibility (Fig. 2). Of them, 563 were excluded, 182 did not undergo any diagnostic studies or refused further evaluations, 147 were diagnosed with UGIB, 100 had post-interventional bleeding, 99 had hemorrhoidal bleeding and 35 underwent a CT protocol not applicable in this study. The final analysis included 382 patients. Of these patients, 112 underwent triple-phase dynamic abdominopelvic MDCT, 65 underwent colonoscopy and 205 underwent sigmoidoscopy as the initial diagnostic test. The baseline characteristics of the enrolled patients are shown in Table 1.

**Table 1:**
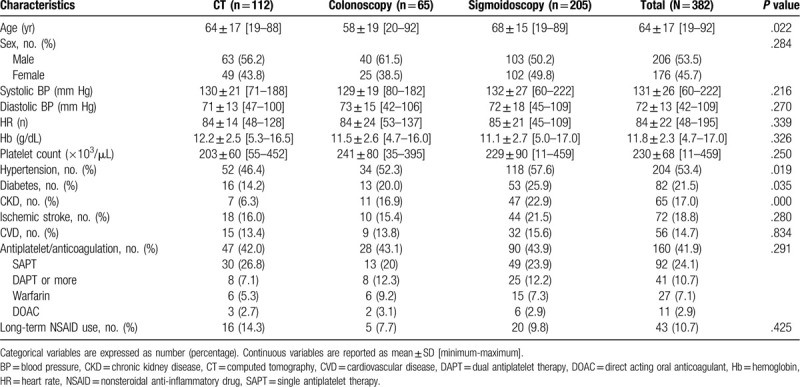
Baseline characteristics.

The diagnostic performances of the initial tests for detecting active bleeding sites are shown in Table 2. CT was initially performed in 112 cases. The initial CT detected the active bleeding sites in 12 cases, while confirmatory tests confirmed that all 12 cases were true active bleeding with no FP diagnosis. The initial CT showed no extravasation of the contrast media or no active bleeding in 100 cases. Among them, 98 cases (98%) were confirmed on the confirmatory tests. In the remaining 2 negative cases (2%), however, the confirmatory tests later revealed active bleeding sites.

**Table 2:**
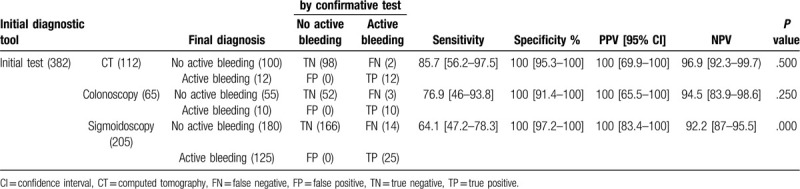
Diagnostic performance of CT, colonoscopy, and sigmoidoscopy for detecting the active bleeding sites in lower gastrointestinal bleeding.

With regard to identifying the etiologies of LGIB, the diagnostic performances of the initial tests are shown in Table 3. When CT was initially performed in 112 cases, 91 cases of LGIB were diagnosed. Among them, 15 cases (16.5%) were later confirmed as false diagnoses. In 21 cases, the CT findings were non-specific, but further evaluations discovered the definite etiologies in 11 cases (52.4%).

**Table 3:**
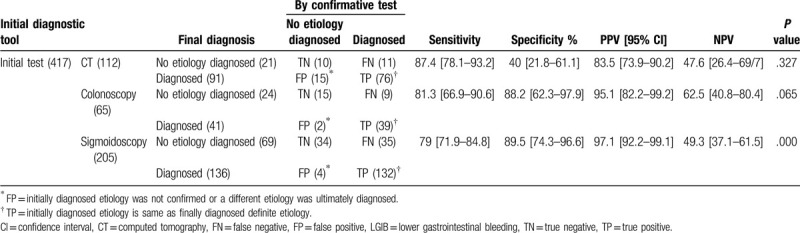
Diagnostic performance of CT, colonoscopy, and sigmoidoscopy for identifying the etiology of lower gastrointestinal bleeding.

Overall, as an initial diagnostic test for the purpose of detecting the active bleeding site, the sensitivity, specificity, PPV, and NPV of CT were high at 85.7%, 100%, 100%, and 96.9%, respectively, and there was no significant difference between the initial CT diagnosis and the final definite diagnosis (*P* = .500). The sensitivity, specificity, PPV, and NPV of colonoscopy for detecting the active bleeding site were similar to those of CT at 76.9%, 100%, 100%, and 94.5%, respectively, and the initial diagnosis was in concordance with the final diagnosis (*P* = .250). Sigmoidoscopy showed high specificity, PPV, and NPV at 100%, 100%, and 92.2%, respectively, but relatively low sensitivity of 64.1% for detecting the active bleeding site, and the initial and final diagnoses were more likely to be non-concordant (*P* = .000).

For diagnosing the etiology of LGIB, the sensitivity, specificity, PPV, and NPV of CT were 87.4%, 40.0%, 83.5%, and 47.6%, respectively (*P* = .327). CT showed high sensitivity and PPV but low specificity and NPV when identifying the etiology causing LGIB. The sensitivity, specificity, PPV, and NPV of colonoscopy for diagnosing the etiology were 81.2%, 88.2%, 95.1%, and 62.5%, respectively (*P* = .065), while those for sigmoidoscopy were 79.0%, 89.5%, 97.1%, and 49.3%, respectively (*P* = .000). The mean admission period and mean time to the final diagnosis were not significantly different among the 3 initial tests (*P* = .915 and .870, respectively) (Table 4).

**Table 4:**
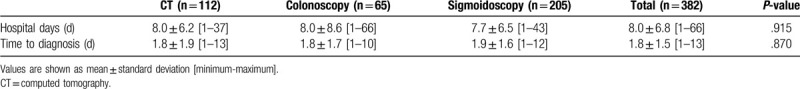
Hospital stay duration and time to final diagnosis.

Of the finally diagnosed etiologies of LGIB, ischemic colitis was the most common underlying etiology (23.8%), followed by colorectal ulcer including Dieulafoy lesion (13.6%), neoplasm (12.3%), diverticular bleeding (11.2%), infectious or non-specific colitis (10.5%), and small bowel bleeding (5.0%). The etiology remained unidentified in 14.7% despite a thorough investigation (Table 5).

**Table 5:**
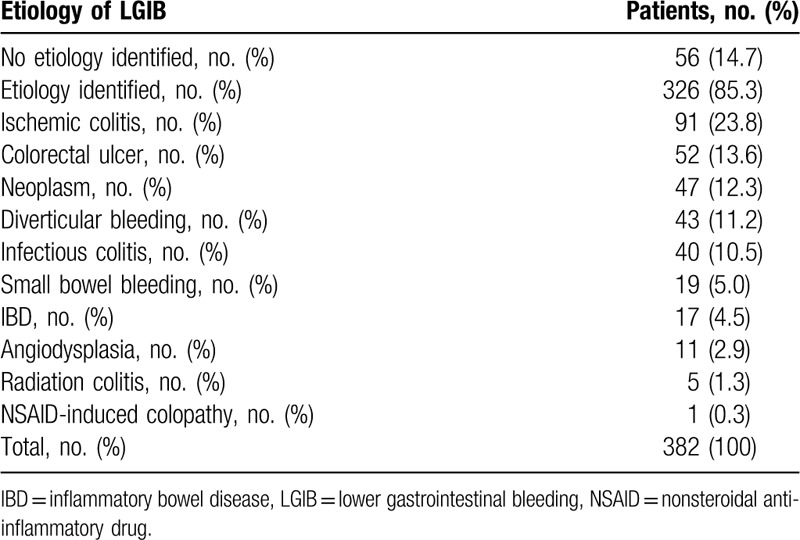
Etiology of LGIB.

Of the 382 LGIB patients, 287 (75.1%) were successfully treated medically. Endoscopic bleeding control, surgery, and embolization were performed in 58 (15.2%), 21 (6.5%), and 12 (3.1%) patients, respectively. The mortality rate of LGIB was 1.83% (n = 7). All of the deceased patients in our study had significant comorbidities other than LGIB.

## 4 Discussion

Considering the difficulties associated with undergoing lower endoscopy in the emergency room and since most cases of LGIB resolve without invasive interventions and show favorable prognosis with a low mortality rate; in many cases, delayed elective colonoscopy will be suitable for LGIB as long as active bleeding is ruled out initially.^[1–4]^ In our study,

A total of 75.1% of patients required conservative care only, with the mortality rate of LGIB being 1.83%, supporting that LGIB mostly shows a benign clinical course. If the patient with LGIB is hemodynamically stable without evidence of active bleeding, emergent colonoscopy is not recommended.^[30]^ Randomized controlled trials have reported that performing urgent colonoscopy in LGIB patients demonstrated no benefit for improving outcomes compared to delayed elective colonoscopy.^[32–34]^ Systemic reviews and meta-analyses by Kouanda et al including 10,172 patients also concluded that there was no clear evidence of the impact of urgent colonoscopy on important clinical outcomes in LGIB patients.^[35]^

We hypothesized that CT would be a good alternative to lower endoscopy as an initial diagnostic tool for evaluating acute LGIB. Unlike colonoscopy, CT is fast, convenient, and non-invasive with easy accessibility. CT may provide more information than endoscopy. CT allows inspection of the entire abdomen including both enteric mucosal and extra-enteric abnormalities. CT is painless and does not require bowel preparation. The major problem of colonoscopy is the low detection rate during active bleeding; in contrast, with CT the faster the bleeding, the more likely the bleeding site will be detected.^[32]^ Kuhle and Sheiman proved in a swine model examination that CTA can depict the extravasation of the contrast media in the lower GI tract with a bleeding rate threshold as low as 0.3 mL/min, which is more sensitive than catheter angiography, which requires a bleeding rate > 0.5 mL/min for detection.^[19,20,36,37]^

If initial CT can accurately detect the active bleeding and etiology of LGIB, this result can guide us to decide whether urgent intervention is necessary or the procedure can be safely delayed. If CT rules out active bleeding, elective colonoscopy may be performed with a proper bowel preparation^[30]^. Furthermore, early localization of the bleeding site by CT would enable targeted intervention.^[19]^ A recent study by Nagata et al concluded that compared to colonoscopy alone, CT before colonoscopy showed a 15.1% increase in the detection rate for vascular lesions.^[22,38]^ Some etiologies are more closely associated with active bleeding and require interventions more often, whereas etiologies such as colitis usually require medical treatment only and early colonoscopy is generally unnecessary or may be even harmful. Lower endoscopy carries the risk of perforation, especially in the presence of severe colitis or diverticulitis. Therefore, early diagnosis of LGIB etiology by CT would lead to safer management of patients.^[39]^

In this retrospective analysis of 382 LGIB patients, CT showed outstanding diagnostic performance at localizing the active bleeding point (sensitivity, specificity, PPV, and NPV of 85.7%, 100%, 100%, and 96.9%, respectively; *P* = .500). Excluding critical active bleeding in advance and early active bleeding site localization were possible with CT. There were no FP findings by CT, meaning that if active bleeding was detected by CT, it was 100% true active bleeding. CT was 100% specific at detecting active bleeding, meaning that CT never misclassified patients without active bleeding as having active bleeding. There were 2 falsely negatively diagnosed cases by CT (1.8%) in which confirmatory tests later revealed active bleeding lesions due to the intermittent nature of some of the LGIB cases. Thus, when no active bleeding point was detected on initial CT, 96.9% of those patients actually did not have active bleeding. Overall, the diagnostic performance of CT at detecting the active bleeding point was outstanding and not inferior to those of colonoscopy and sigmoidoscopy. Upon detecting the extravasation of the contrast media on the initial CT, immediate management should be considered. If no extravasation is detected on the initial CT, no critical active bleeding would likely be present, so performing delayed colonoscopy would be possible.

Colonoscopy was accurate for detecting active bleeding (sensitivity, specificity, NPV, and PPV of 76.9%, 100.0%, 100%, and 94.5%, respectively; *P* = .250). Among the initial 65 colonoscopic diagnoses, there were 3 FN results; all were small bowel bleeding lesions that could not be localized by colonoscopy and were later discovered by CT, supporting CT's usefulness. Sigmoidoscopy was the most widely used initial diagnostic tool at the emergency room in our institution (n = 205), but it was the least accurate initial tool for detecting active bleeding and featured low sensitivity. In 10 of 14 initial FN sigmoidoscopic cases, CT later revealed the active bleeding site, supporting CT's diagnostic ability.

CT showed some limitations for diagnosing the exact etiology of LGIB due to its low specificity (40.0%) and NPV (47.6%), but it still showed high sensitivity (87.4%) and PPV (83.5%). While the initial CT did not diagnose the etiology in 21 of 112 total CT cases, later tests revealed a definite diagnosis in 11 of 21 undiagnosed cases, resulting in NPV of 47.6%. However, the etiologies of LGIB not detected on the initial CT showed very favorable clinical outcomes. None were associated with active bleeding and required endoscopic or radiological interventions. In all cases, the bleeding stopped spontaneously with only supportive care. In addition, CT showed a PPV of 83.5% when diagnosing the etiology, meaning that an initially diagnosed etiology by CT had an 83.5% chance of being concordant with the final confirmed true etiology.

The mean admission period and mean time to the final diagnosis were not extended when CT was used as an initial test instead of lower endoscopy, which further supports that CT is not inferior to lower endoscopy as a primary diagnostic tool.

The strength of our study is that, compared with previously published studies assessing the diagnostic ability of CT for LGIB, our analysis included a larger number of cases over a longer study period.^[18,19,21,22,40,41]^ To our knowledge, our study included the largest sample of LGIB patients undergoing CT or lower endoscopy. The limitations include that our analysis was a single-center study and selection bias may be present due to its retrospective design. In addition, when reading the CT images, the radiologist was not blinded to the patients’ clinical information, which might have influenced the CT reading results. This study only included the triple-phase dynamic abdominopelciv MDCT as an initial test. CTA protocol was not included. Reis et al reviewed various CT and CTA protocols for evaluating GI bleeding and found out that definitions of CT and CTA protocols were very diverse across the centers.^[24]^ There was no clear consensus regarding which combination of parameters (phases, concentration, dose, volume and injection rate of intravenous contrast media, tube voltage, abdominal aorta threshold, etc) would yield the best diagnostic performance for LGIB, while avoiding unnecessary procedures.^[24]^ Further research may be necessary to determine the most optimal CT protocol for diagnosing LGIB.

In conclusion, our findings demonstrated that CT was not inferior to lower endoscopy as an initial diagnostic tool for evaluating LGIB patients presenting as hematochezia in an emergency room. CT can be recommended as a rapid and convenient initial diagnostic tool instead of colonoscopy or sigmoidoscopy for evaluating acute LGIB. CT is highly accurate and not inferior to lower endoscopy for detecting the active bleeding site. Early accurate localization and the exclusion of potentially critical active bleeding is possible with CT. Also, the etiology can be diagnosed with high sensitivity and PPV by CT. Based on the information provided by initial CT, the most appropriate treatment plan can be planned in advance. Compared to initial lower endoscopy, initial CT did not extend the hospital stay or time to the definite diagnosis. Thus, we propose the use of CT as a first-line diagnostic tool for evaluating cases of acute LGIB.

## Author contributions

**Conceptualization:** Sun Hyung Kang, Ju Seok Kim.

**Data curation:** Hee Sung Lee, Eaum Seok Lee.

**Formal analysis:** Hee Sung Lee, Sun Hyung Kang, Eaum Seok Lee.

**Investigation:** Woo Sun Rou, Hyuk Soo Eun, Ju Seok Kim, Hee Seok Moon, Jae Kyu Sung.

**Methodology:** Sun Hyung Kang, Woo Sun Rou, Jong Seok Joo, Hee Seok Moon, Jae Kyu Sung.

**Project administration:** Hyuk Soo Eun.

**Software:** Seok Hyung Kim.

**Supervision:** Sun Hyung Kang, Seok Hyung Kim, Byung Seok Lee, Hyun Yong Jeong.

**Validation:** Jong Seok Joo.

**Writing – original draft:** Hee Sung Lee.

**Writing – review & editing:** Sun Hyung Kang.
